# Gold Nanoparticle-Assisted Virus Formation by Means of the Delivery of an Oncolytic Adenovirus Genome

**DOI:** 10.3390/nano10061183

**Published:** 2020-06-17

**Authors:** Luis Sendra, Antonio Miguel, M. Carmen Navarro-Plaza, María José Herrero, José de la Higuera, Consuelo Cháfer-Pericás, Elena Aznar, M. Dolores Marcos, Ramón Martínez-Máñez, Luis Alfonso Rojas, Ramón Alemany, Salvador F. Aliño

**Affiliations:** 1 Gene Therapy Group, Department of Pharmacology, Faculty of Medicine, Universitat de València, Av Blasco Ibáñez 15, 46010 València, Spain; luis.sendra@uv.es (L.S.); matasantonio@hotmail.com (A.M.); marinp_itq@hotmail.com (M.C.N.-P.); jdlhadalid@gmail.com (J.d.l.H.); 2 Pharmacogenetics Unit, Instituto de Investigación Sanitaria La Fe, Av Fernando Abril Martorell 106, 46026 València, Spain; 3 Instituto Interuniversitario de Investigación de Reconocimiento Molecular y Desarrollo Tecnológico, Universitat Politècnica de València, Universitat de València, Camino de Vera s/n, 46022 Valencia, Spain; m.consuelo.chafer@uv.es (C.C.-P.); mmarcos@qim.upv.es (M.D.M.); rmaez@qim.upv.es (R.M.-M.); 4 CIBER de Bioingeniería, Biomateriales y Nanomedicina (CIBER-BBN), 28029 Madrid, Spain; 5 Unidad Mixta de Investigación en Nanomedicina y Sensores, Universitat Politècnica de València, Instituto de Investigación Sanitaria La Fe, Av Fernando Abril Martorell 106, 46026 Valencia, Spain; 6 Catalan Institute of Oncology–IDIBELL, Av Gran Via 199-203, 08908 Barcelona, L’Hospitalet de Llobregat, Spain; rojasexl@mskcc.org (L.A.R.); ralemany@iconcologia.net (R.A.); 7 Clinical Pharmacology Unit, Drug Clinical Area, Hospital Universitario y Politécnico La Fe, Av Fernando Abril Martorell 106, 46026 València, Spain

**Keywords:** gold nanoparticles, delivery, gene therapy, non-viral vectors, oncolytic virus, virotherapy, cancer

## Abstract

Oncolytic adenoviruses are a therapeutic alternative to treat cancer based on their ability to replicate selectively in tumor cells. However, their use is limited mainly by the neutralizing antibody (Nab) immune response that prevents repeated dosing. An alternative to facilitate the DNA access to the tumor even in the presence of anti-viral Nabs could be gold nanoparticles able to transfer DNA molecules. However, the ability of these nanoparticles to carry large DNA molecules, such as an oncolytic adenovirus genome, has not been studied. In this work, gold nanoparticles were functionalized with different amounts of polyethylenimine to transfer in a safe and efficient manner a large oncolytic virus genome. Their transfer efficacy and final effect of the oncolytic virus in cancer cells are studied. For each synthesized nanoparticle, (a) DNA loading capacity, (b) complex size, (c) DNA protection ability, (d) transfection efficacy and (e) cytotoxic effect were studied. We observed that small gold nanoparticles (70–80 nm in diameter) protected DNA against nucleases and were able to transfect the ICOVIR-15 oncolytic virus genome encoded in pLR1 plasmid. In the present work, efficient transgene RNA expression, luciferase activity and viral cytopathic effect on cancer cells are reported. These results suggest gold nanoparticles to be an efficient and safe vector for oncolytic adenovirus genome transfer.

## 1. Introduction

The use of nanoparticles (NPs) for transferring nucleic acids has attracted increasing interest due to their low cytotoxicity, immunogenicity, biocompatibility and the easy functionalization of their surface [1,2,3]. Previous studies have shown the efficacy of gold nanoparticles (AuNPs) as DNA carriers [4,5,6,7,8]. Thanks to their physico-chemical features, AuNPs can be decorated with cationic molecules to control the surface charge of nanostructures and facilitate DNA binding by an electrostatic interaction [9,10]. Specifically, it has been demonstrated that the chemical functionalization of AuNPs with the cationic polymer polyethylenimine (PEI) improves DNA-binding and enables “in vitro” transfection [5]. Cationic groups promote electrostatic interactions with the negative charges of DNA, allowing the formation of complexes that can be internalized by cells. Several studies confirm that positively charged NPs exhibit greater affinity for cell membranes than the negatively charged ones, being more efficient at entering the cytoplasm. This is an important consideration in applications involving the transfer of biomolecules into the cell nucleus [11]. Following these procedures, AuNPs have proven their efficacy in several gene therapy applications [12,13,14,15,16,17,18,19,20,21,22,23]. However, the ability of these nanoparticles to carry large DNA molecules, such as an oncolytic adenovirus genome, has not been studied so far. Oncolytic viruses can selectively destroy cancer cells and have already proved to be efficient in some tumors. Several oncolytic viruses have proven their efficacy and tolerability in clinical trials [24,25,26,27,28,29]. In this sense, an oncolytic virus to treat neck and head cancer (H101, Oncorine^®^, modified adenovirus) was approved in China in 2006, and another one to treat advanced melanoma (T-VEC, Imlygic^®^, modified herpes simplex virus-1 expressing GM-CSF) was approved by the FDA and EMA in December 2015. However, the use of oncolytic viruses is partially limited because of their immunogenicity and the pre-existing humoral immunity in the general population [30,31,32] or the humoral immunity elicited by the first virus administration in repeated doses, which is able to neutralize their infectivity. As only systemic therapeutics can target both primary and secondary tumors, recent studies have focused on the development of carriers for oncolytic adenoviruses delivery to tumor tissues via a systemic route [33], such as hybrid vector systems generated by complexing oncolytic Ad with nanoparticles [34]. Employing plasmids that encode oncolytic viruses instead of using viruses themselves could mediate a conditional replication of the virus only in cancer cells but avoid their undesired immune effect [35]. This could permit employing these viral genome plasmids upon repeated administration for longer periods without losing their efficacy. A study in mice showed a promising antitumor effect using an oncolytic plasmid encoding the adenovirus death protein [36]. In this sense, and aiming at avoiding the use of oncolytic adenovirus itself and its immunogenicity, we have developed PEI-AuNPs that allow carrying large DNA molecules, such as the adenovirus genome. This strategy avoids the neutralization of the therapeutic agent by circumventing the use of an immunogenic viral particle and by coating the DNA with nanoparticles to prevent the nucleases’ action.

In this work, AuNPs were functionalized with different PEI ratios to identify the optimal conditions that permit carrying the maximum amount of DNA (DNA/PEI-AuNPs weight ratio of 2.5:1) with the smallest complex size (around 70 nm). Nanoparticles were physico-chemically characterized. Further, their toxicity in different cell lines and their ability to protect DNA was assessed. Finally, these nanoparticles were complexed with pLR1 plasmid, which encodes the oncolytic adenovirus ICOVIR-15 with proved tolerability and efficacy in a murine model [37], and were transfected into human tumor cells. The viral RNA copy number was determined at different sampling time points and the viral replication and oncolytic capacity were evaluated.

## 2. Materials and Methods

### 2.1. Materials and Reagents

#### 2.1.1. Chemicals

PEI (25 kDa), hydrogen tetrachloroaurate (III), sodium borohydride, 2-pyridyldithiol-tetraoxatetradecane-N-hydroxysuccinimide (SPDP), dithiothreitol (DTT), HEPES, NaCl and NaOH were purchased from Sigma-Aldrich (St. Louis, MO, USA). HBS buffer was prepared from 20 mM HEPES and 150 mM NaCl, and adjusted to pH 7.4 with NaOH. Ethanol was obtained from Scharlab (Barcelona, Spain). All the reagents were used as received without further purification. PD-10 columns were obtained from VWR (Radnor, PA, USA). The dialysis membrane (7-Spectra/Por, MWCO: 50,000) was purchased from Spectrum Chemical Mfg. Corp. (Gardena, CA, USA).

#### 2.1.2. Plasmids

The plasmids used in this work were p3C-eGFP, p2F-hIL10, pAdZhcICOVIR15-fi-Luc (pLR1) and pVK503TL. The plasmid p3C-eGFP (6.45 kb), containing the enhanced green fluorescent protein (eGFP) complementary DNA (cDNA) driven by the CMV (cytomegalovirus) promoter, was constructed by cloning eGFP cDNA into the HindIII site of pcDNA3 (Invitrogen, Waltham, MA, USA), excised from the peGFP-N1 (4.7 kb) plasmid vector (Clontech Laboratories, Saint-Germain-en-Laye, France). Plasmid pAdZhcICOVIR15-fi-Luc or pLR1 (42 kb) contains DNA encoding the ICOVIR-15Luc oncolytic adenovirus [38] which expresses luciferase under the major late promoter (whose activation depends on virus replication), and contains a plasmid backbone formed by a replication origin, ampicillin resistance gene and endonuclease I-SceI gene. pLR1 is a self-excising plasmid. Arming an oncolytic adenovirus with a splicing acceptor connected to the MLP renders an expression dependent of virus replication [39].

When a eukaryotic cell is transfected, I-SceI nuclease is expressed and breaks its targets, flanking the adenoviral genome, and linearizing it [40]. The pVK503TL plasmid has the same size as pLR1 and contains the GFP and luciferase genes [41].

#### 2.1.3. Cell Lines

The cell lines used for this study were B16F10 (B16), HepG2 and SW480 and were obtained from ATCC^®^ (Manassas, VA, USA). B16 cells were derived from a murine melanoma tumor. HepG2 cells were derived from a human hepatocellular carcinoma and SW480 cells were derived from a human colorectal adenocarcinoma. Cells were maintained in Dulbecco’s modified Eagle’s medium (DMEM; Sigma-Aldrich, St Louis, MO, USA) supplemented with 10% inactivated fetal bovine serum (HyClone^®^, Thermo Scientific, Waltham, MA, USA) and 1% antibiotics (100 U/mL penicillin, 100 m.u.g/mL streptomycin) (Biochrom, Cambridge, UK). Cells were incubated at 37 °C in a humidified atmosphere containing 5% CO_2_ in 25, 75 or 175 cm^2^ flasks (Corning-Fisher scientific, Corning, New York, NY, USA), as required.

### 2.2. Synthesis of Nanoparticles and Complexes

#### 2.2.1. PEI-AuNPs

The synthesis of PEI-AuNPs with diameters <10 nm was carried out at room temperature using PEI both as the stabilizer and functionalizing agent following the method described by Brust et al. [42]. Firstly, PEI was derivatized with thiol groups (PEI-SH) using the procedure described by Aliño et al. [43]. In particular, 40 µL of SPDP (20 mM ethanol) was added to 2.5 mL of the corresponding PEI solution (1, 2, 4, 8 mg/mL in HBS, pH: 7.4). The mixture was incubated and stirred for 30 min. The resulting product (PEI-PDP) was purified in a PD-10 column, using HBS as the eluent.

Disulfide bridges of the PEI-PDP product can be cleaved by adding 12 µL of DTT (1 M) per 0.5 mL of sample giving the product PEI-SH. DTT addition ruptures the disulfide bonding of the pyridyldithiopropionate (PDP) groups, releasing one 2-thiopyridine (DTP) molecule per PDP linker. To determine the number of thiol groups in the final PEI-SH product, the absorbance of the aqueous solution containing PEI-PDP was measured at 343 nm before and after the addition of DTT. As the DTP extinction coefficient is 8080 M^−1^ cm, the DTP concentration, and thus also the PDP concentration in each sample were calculated from the expression (absorbance at λ_343_)/8080 [44]. The obtained thiolated PEI (PEI-SH) was purified in a PD-10 column, using HBS as the eluent.

To prepare the nanoparticles, PEI-SH solution was added to a solution of HAuCl_4_ (15 mL, 1 mM in deionized water) under vigorous stirring for 10 min. Then, gold reduction was performed by adding an aqueous solution of sodium borohydride (NaBH_4_ 0.4 M) dropwise until the molar concentration of NaBH_4_ was 20-fold higher than the Au salt concentration. The mixture was stirred vigorously for 24 h. The PEI-AuNPs obtained were purified by dialysis in deionized water for 24 h employing a 50 KDa cut-off membrane (7-Spectra/Por, MWCO: 50,000) purchased from Spectrum Chemical Mfg. Corp. (Gardena, CA, USA), aiming to be sure that nanoparticles were retained in the dialysis tube and all unbounded PEI was removed. To assure the elimination of all free PEI, the aqueous solution was changed every 3 h during the first 12 h of dialysis.

#### 2.2.2. PEI-AuNPs/DNA Complexes

To prepare the PEI-AuNPs/DNA complexes, a suspension containing 250 µL of HBS (pH = 7.4) and 250 µL of nanoparticles was prepared. Then, 500 µL of a second solution containing eGFP plasmid in HBS was added to the AuNPs suspension and shaken vigorously. Different suspensions varying in the amount of DNA were prepared in the range of the Au-NPs/DNA weight ratio of 1:0 01 to 1:2.5 µg/µg (1:0.01; 1:0.07; 1:0.3; 1:0.6; 1:1.25 and 1:2.5). Finally, the mixtures were incubated for 30 min to allow the electrostatic binding of DNA to the surface of the nanoparticles.

### 2.3. Characterization of PEI-AuNPs and PEI-AuNPs/DNA Complexes

The size distribution and zeta potential of the NPs and NPs/DNA complexes were determined using the particle size analyzer Malvern Zetasizer Nanoseries-Nano ZS (Malvern, UK). Morphological studies of the different NPs were performed by transmission electron microscopy (TEM) using a 100 kV Philips CM 10 microscope (Hillsboro, OR, USA), working at 100 kV. The amount of PEI polymer attached to the NPs surface was evaluated by thermogravimetric analysis on a TGA/SDTA 851e Mettler Toledo balance (Columbus, OH, USA). To study the stability of the DNA attached to the PEI-AuNPs from the nuclease degradation, the PEI-AuNP/DNAs were incubated in the presence or absence of serum (25%). Furthermore, the DNA-PEI-AuNPs were incubated with or without heparin to study the dissociation rate of the complex, as previously described [45]. After this incubation, each sample was run on 0.8% agarose gel with the RedSafe DNA marker (Fisher Scientific, Waltham, MA, USA) for electrophoretic separation and purification. Finally, the gel was revealed with a UV light trans-illuminator.

### 2.4. Transfection

#### 2.4.1. Transfection Procedure

Cells were transfected by a chemical procedure based on PEI 25 kDa (polyethyleneimine, Sigma, St Louis, MO, USA) polyplexes (*w*/*w* DNA:PEI ratio of 1:1.41), as previously described [42], but using AuNPs-PEI instead of PEI. Twelve-well plates were employed and the seeding quantity was established in 1 × 10^5^ cells per well. Cells were transfected during their exponential growth phase, when the confluence was higher than 80% in the flasks, determined de visu by light transmission microscopy. In brief, PEI/DNA complexes are formed by mixing them in the DNA/PEI molar ratio of 1:1.41. These complexes were added with serum-free medium to cultured cells, incubated for 1 h at 37° and 5% CO_2_ and then, the culture medium supplemented with serum was added. Culture media were exchanged every 48 h. Once treated, cells were not passaged. Only floating cells were removed when media were exchanged.

#### 2.4.2. Evaluation of AuNPs-eGFP Transfection

The transfection efficiency was determined by evaluating the expression of eGFP protein encoded by the gene construct, compared with that observed in controls transfected by PEI/DNA polyplexes. For this purpose, the fluorescence of eGFP protein was determined by observing the transfected cells under a fluorescence microscope (Axiovert 135M, Zeiss, Oberkochen, Germany) 24 and 48 h after transfection. Fluorescence intensity was also quantified using a microplate fluorimeter (Millipore Cytofluor 2300, Burlington, MA, USA).

### 2.5. Transfection of Nanoparticles Carrying Large DNA (pLR1 Plasmid)

#### 2.5.1. Nanoparticles Characterization

The plasmid pLR1 was added to PEI-AuNPs at different weight ratios of PEI-AuNPs/pLR1: 1:0.0375–1:2.5. Then, the size of the resulting PEI-AuNPs/pLR1 complexes was measured by a Zetasizer analyzer, as described above.

#### 2.5.2. Evaluation of AuNPs Efficacy for Large DNA Transfection

HepG2 and SW480 cells were transfected with PEI-AuNPs/pVK503TL employing 5, 10 or 20 µg/mL of pVK503TL at a PEI-AuNPs/pVK503TL ratio of 1:2.5 (µg/µg). Expression of pVK503TL was evaluated 48 and 72 h after transfection by determining the fluorescence emitted by cells in a fluorimeter (Millipore Cytofluor 2300, Burlington, MA, USA), and by observing the cell culture fluorescence under the microscope (Axiovert 135M, Zeiss, Oberkochen, Germany).

#### 2.5.3. Transfection Efficacy of AuNPs/pLR1

The efficiency of PEI-AuNPs/pLR1 transfection was evaluated by quantifying the copy number of plasmid DNA present in the cell culture at different time points (1, 3, 7, 10, 14, 21 and 28 days) after transfection. This was performed with real-time PCR.

### 2.6. Real Time PCR

RNA was obtained from cultured cells with a commercial kit (RNeasy Mini Kit [Qiagen NV, Venlo, the Netherlands]), following manufacturer instructions. Prior to the nucleic acid extraction and purification, the culture medium was removed and cells were washed with PBS. Only the pellet of cells was employed for the nucleic acid extraction. During RNA purification, DNA was completely removed by DNAse incubation to assure that all the possible remaining plasmid was degraded. Quantitative PCR using a 7900HT Fast Real-Time PCR System (Thermo Fisher Scientific) allowed the quantitation of the plasmid RNA copy number. RNA was measured from cell lysates and the number of RNA copies was obtained plotting the results on a standard curve prepared with known amounts of the same pLR1 plasmid, that permitted us obtaining the absolute quantification of the pLR1 RNA presence. To amplify the RNA, specific primers and SYBR Green Master Mix were used. Primers were designed to determine the Ad5 hexon gene (in L3 transcription unit). They amplify the sequence corresponding to nt 18852 to nt 18918 of the Ad5wt reference sequence.

Fw: Ad18852 5′-CTTCGATGATGCCGCAGTG-3′

Rv: Ad18918R 5′-GGGCTCAGGTACTCCGAGG-3′

### 2.7. Cell Viability Assay

To evaluate the cell toxicity mediated by the nanoparticles, we used the calcein esters metabolization test (calcein-AM, Invitrogen, Waltham, MA, USA). Cell viability was assessed 24, 48 and 72 h after transfection as follows: calcein-AM was added to the cell culture for a final 1.25 mM concentration in the well and, 40 min later, the fluorescence was measured with a fluorimeter (Millipore Cytofluor 2300, Burlington, MA, USA).

## 3. Results

### 3.1. Characterization of PEI-AuNPs

**Figure 1:**
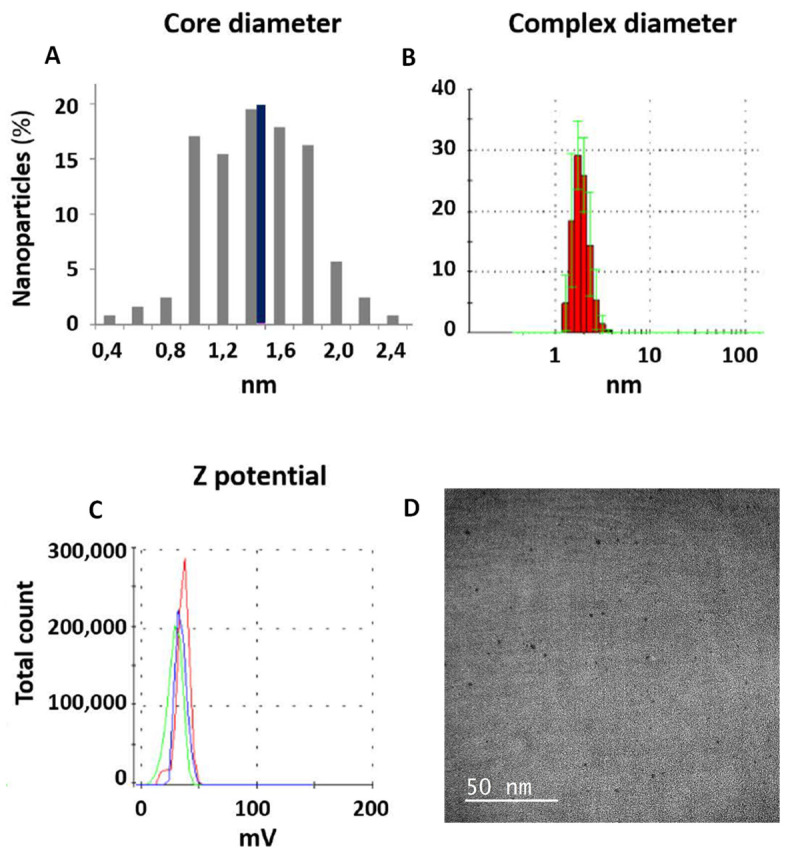
PEI-Au NPs synthesized. (**A**) Core diameter distribution obtained from the TEM data for the nanoparticles synthesized with an 8 mg/mL PEI solution (NP4). Black bar indicates the average size ≈ 1.5 nm. (**B**) Analysis of NP4 hydrodynamic diameter ≈ 2.10 nm. Measurements made by the particle size analyzer to the NPs synthesized with a solution of 8 mg/mL of PEI (NP4). (**C**) NP4 zeta potential ≈ 34 mV. (**D**) Representative TEM image of NP4.

AuNPs were synthesized in the presence of different concentrations of the cationic PEI polymer (1 mg/mL, NP1; 2 mg/mL, NP2; 4 mg/mL, NP3; and 8 mg/mL, NP4). NPs’ core diameters were measured by TEM and their hydrodynamic diameters were measured by a Zetasizer. The zeta potential of the PEI-AuNPs was also determined for each one of the PEI concentrations employed (Table 1). The size of the PEI-AuNPs decreases proportionally as the amount of PEI increases. Employing 1 mg/mL of PEI, the hydrodynamic diameter achieved was 86.6 nm, probably due to the low interaction between the PEI chains and the low possibility of one DNA molecule to interact with the different PEI. NPs 2 and 3 mediated a better reduction in the hydrodynamic diameter, which was reduced to 6.5 and 5.5 nm, respectively. The concentration of PEI that offered the minimum nanoparticle core (1.5 nm) and hydrodynamic diameter (2.1 nm) was 8 mg/mL (NP4), which also had the highest positive charge and permitted carrying the maximum DNA amount with the minimum nanoparticle size (Figure 1). For this reason, NP4 was selected for the gene transfections.

### 3.2. Characterization of AuNPs/DNA COMPLEX USINg the p3CeGFP Small Plasmid

NP4 nanoparticles were used to form PEI-AuNPs/DNA complexes through an electrostatic interaction, using the p3CeGFP plasmid. Different complexes were formed by adding increasing amounts of DNA (p3C eGFP) to a constant amount of nanoparticles and incubating them for 30 min at 37 °C. The weight ratios of the AuNPs-PEI/DNA employed ranged from 1:0.01 to 1:2.5. After the complexes formation, their size and Z potential were determined. Table 2 includes information regarding the complexes’ diameters and Z potential varying with the amount of DNA added. As it can be shown in the table, the size is higher as the amount of DNA increases, remaining constant between 1:0.03 to 1:1.25 ratios. The weight ratio of PEI-AuNPs/p3CeGFP that allowed carrying the largest amount of DNA per nanoparticle was 1:2.5. This results in NPs with a hydrodynamic diameter of approximately 70 nm. This condition was the one employed in next experiments.

### 3.3. Transfections with PEI-AuNPs/p3C eGFP

**Figure 2:**
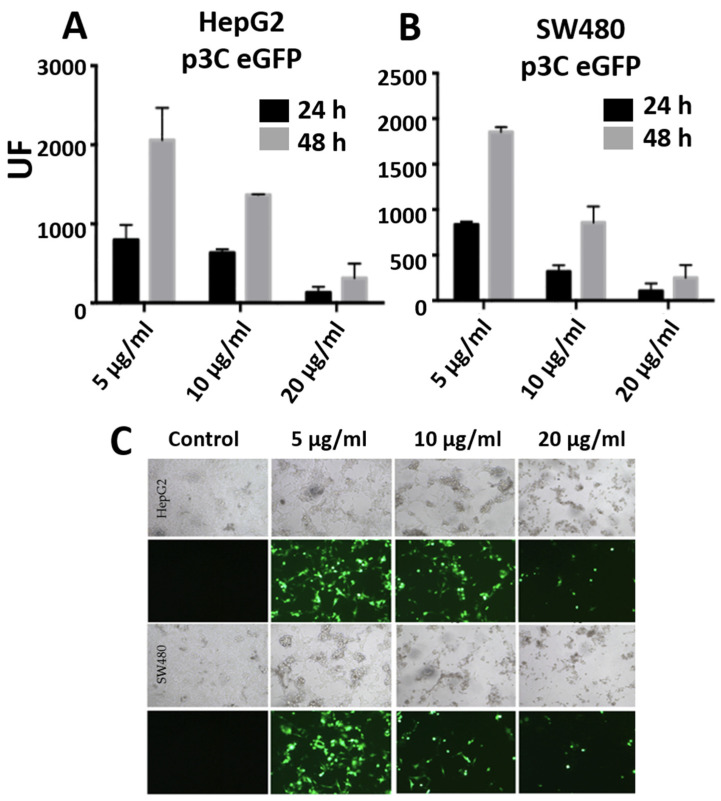
Graphs (**A**,**B**) represent fluorescence of HepG2 and SW480, respectively, 24 and 48 h after transfection with PEI-AuNPs/p3C eGFP with 5, 10 or 20 µg/mL of p3C eGFP at a relation of PEI-AuNPs/p3C eGFP of 1:2.5 (µg/µg). Figure (**C**) shows the fluorescence microscope images of HepG2 and SW480 cells 48 h after transfection with PEI-AuNPs/p3C eGFP at 1:2.5 (µg/µg) using different DNA concentrations. Images were captured at 20× magnification.

The DNA transfection efficiency of gold nanoparticles bearing the plasmid p3CeGFP (which encodes the eGFP fluorescent protein) was measured. Figure 2 shows the fluorescence intensity 24 and 48 h after HepG2 (Figure 2A) and SW480 (Figure 2B) cells’ transfection with PEI-AuNPs/p3CeGFP. The maximum intensity of fluorescence was observed 48 h after transfection and corresponded with the 5 µg/mL DNA concentration. The fluorescence observed was higher in the HepG2 than in the SW480 cells. Figure 2C shows the fluorescence microscopy images of GFP expression 48 h after transfection at 5, 10 and 20 µg/mL of p3CeGFP. The highest percentage of fluorescent cells was achieved with 5 µg/mL p3CeGFP concentration. Progressive cell toxicity was observed as the DNA and PEI doses increased from 5 µg/mL.

### 3.4. AuNPs Protection of DNA against Nucleases

**Figure 3:**
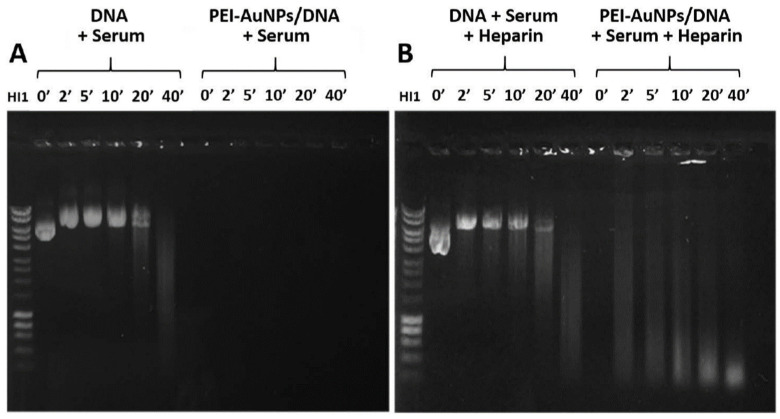
Protection of DNA nuclease degradation. These electrophoresis gels show samples of DNA (p3C eGFP) or PEI-AuNPs/DNA incubated during different times in the presence of serum (**A**) or serum + heparin (**B**). The relation of PEI-AuNPs/p3C eGFP used was 1:2.5 (µg/µg).

In order to evaluate the potential use of AuNPs in vivo, the protection of DNA bound to the PEI (8 mg/mL)-AuNPs against circulating nucleases was studied. The complexes were incubated with 25% mice serum in the absence (Figure 3A) and presence (Figure 3B) of heparin, which can dissociate the PEI-AuNPs/DNA complex. After the different incubation times, the samples were removed and ran on 0.8% agarose gel. Figure 3A shows that whereas the DNA complexed with PEI-AuNPs remained retained in the wells and was not degraded in the presence of serum, free DNA was progressively degraded after 20–40 min of incubation. The lack of fluorescence is due to the quenching effect caused by gold nanoparticles when forming the compact complexes generated [46]. This suggests that nanoparticles protect DNA against serum nucleases. However, when PEI-AuNPs/DNA complexes were incubated in the presence of polyanionic heparin (Figure 3B), the DNA dissociated from PEI-AuNPs, being degraded by serum nucleases. Total degradation of free DNA in the presence of serum with heparin can be observed at 40 min of incubation. In these conditions, the degradation of DNA from the PEI-AuNPs complexes is unexpectedly faster (visible from 2 min of serum incubation) than in free DNA. 

### 3.5. Characterization of PEI-AuNPs/DNA Using an Oncolytic Adenovirus Genome Plasmid

**Figure 4:**
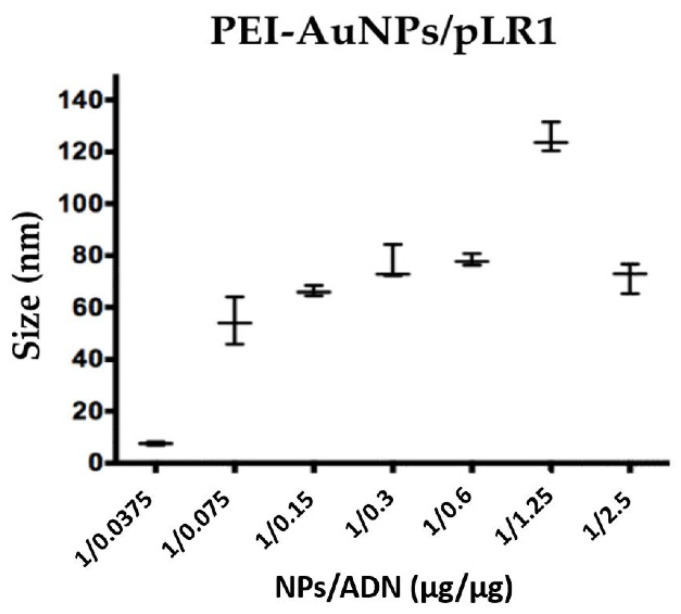
Size of PEI-AuNPs/pLR1. This graph represents the size of the PEI-AuNPs/pLR1 at different weight ratios of PEI-AuNPs and DNA (pLR1) measured by a Zetasizer. The PEI-AuNPs used are NP4 that were synthesized with 8 mg/mL of the initial concentration of PEI.

After confirming that AuNPs were able to protect and effectively transfect p3CeGFP plasmid in the cells, we studied whether they could transfect cells with a larger plasmid encoding an oncolytic virus. The plasmid used for these assays was pLR1, which encodes the ICOVIR-15Luc oncolytic virus expressing the luciferase gene. When transfected, this plasmid expresses the I-Sce restriction enzyme that excises the complete genome of the virus. Upon excision, the genome is able to replicate and to generate viral particles. Luciferase is expressed as a splicing unit connected to the major late promoter and therefore is only expressed upon virus replication. Figure 4 represents the complexes’ size (evaluated by Zetasizer) for each one of the PEI-AuNPs/pLR1 weight ratios assayed: 1:0.0375–1:2.5. The most interesting weight ratio was 1:2.5 since it generated complexes with a diameter around 70 nm, with the ability of carrying a great amount of DNA.

### 3.6. Transfections with Large Plasmids: PEI-AuNPs/pVK503TL and PEI-AuNPs/pLR1

**Figure 5:**
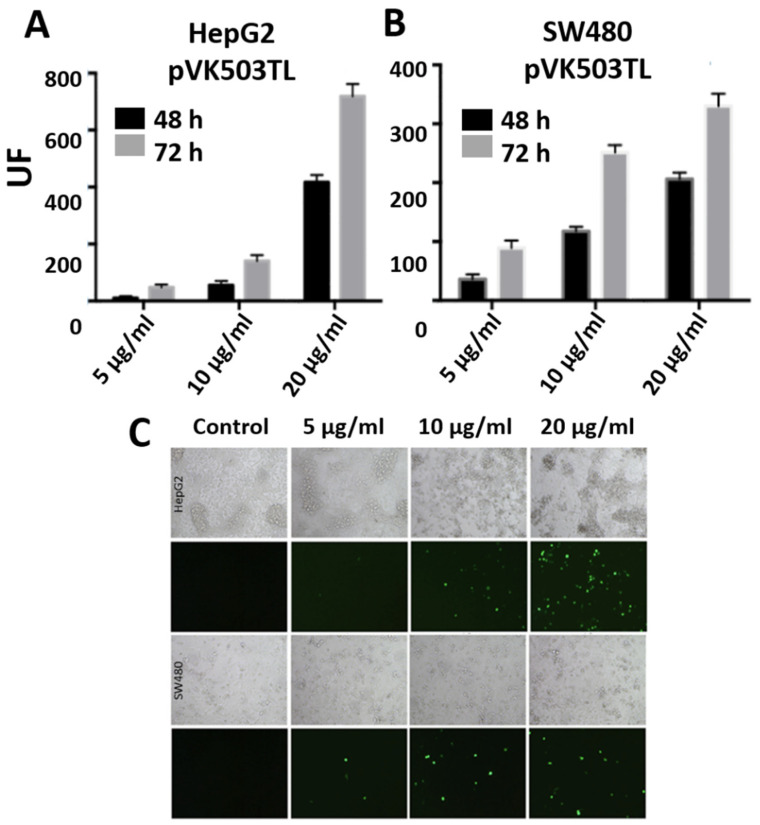
Graphs (**A**,**B**) represent the fluorescence of HepG2 and SW480, respectively, 48 or 72 h after transfection with PEI-AuNPs/pVK503TL with 5, 10 or 20 µg/mL of pVK503TL at a relation of PEI-AuNPs/pVK503TL of 1:2.5 (µg/µg). Figure (**C**) shows the comparative transmission and fluorescence microscope images of HepG2 and SW480 cells 48 h after transfection with PEI-AuNPs/pVK503TL at 1:2.5 (µg/µg) using different DNA concentration. Images were captured at 20× magnification.

In order to select the optimal DNA concentration to transfect cells with nanoparticles and large plasmids, the pVK503TL reporter plasmid, which has the same size as pLR1 and encodes two expression cassettes, for GFP and luciferase genes, substitutes the E1 region of the adenovirus genome. These cassettes are expressed under the CMV promoter when the virus genome reaches the nucleus of the transfected cells, independently of virus replication. The plasmid is not self-slicing and therefore it does not generate viruses upon transfection unless the virus genome is released previously (using flaking PacI sites). Figure 5A,B represents the fluorescence intensity achieved in HepG2 and SW480 cells 48 and 72 h after transfection with PEI-AuNPs/pVK503TL with a 1:2.5 weight ratio. The highest expression of GFP was observed in both cell lines using 20 µg/mL of DNA. Figure 5C shows the fluorescence microscopy images of GFP expression 72 h after transfection with each DNA concentration assayed.

**Figure 6:**
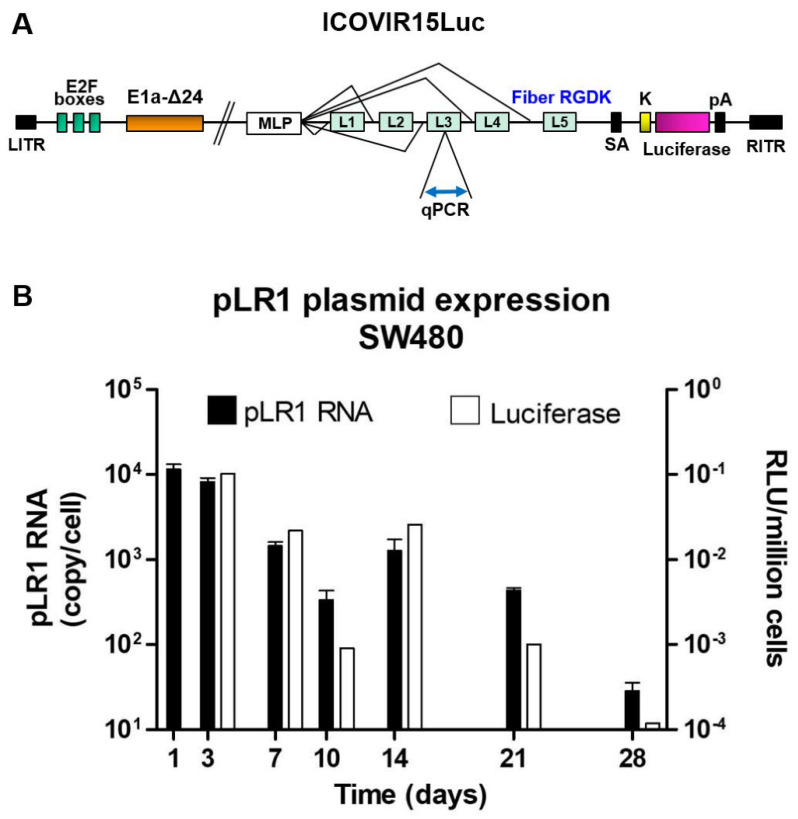
(**A**) Scheme of ICOVIR15Luc encoded by pLR1 plasmid. qPCR indicates the location of the target sequence for the quantitative polymerase chain reaction on the Ad5 hexon gene, present within late transcription unit 3 (L3). LITR: left inverted terminal repeat; E2F boxes: locations for E2F transcription factors; E1A: early transcription unit 1; MLP: major late promotor; L1–L5: late transcription units; SA: splicing acceptor; K: replacement of KKTK motif with RGDK; pA: polyadenylation signal sequence; RITR: right inverted terminal repeat. (**B**) Graph represents RNA copies of pLR1 (black bars) at different days after transfection of SW480 cells with PEI-AuNPs/pLR1 at 20 µg/mL of pLR1. RNA was measured from cell lysates and results were plotted on a standard curve prepared with known amounts of the same plasmid. White bars (plotted on right Y axis) represent luminescence at different days of samples from the SW480 cells transfected with PEI-AuNPs/pLR1 at 20 µg/mL of pLR1.

Once it was observed that the nanoparticles were able to transfect the large pVK503TL plasmid, we transfected the cells with pLR1 plasmid (which encodes ICOVIR-15Luc, schemed in Figure 6A). We used the optimal 20 µg/mL concentration of pLR1 and a PEI-AuNPs/pLR1 weight ratio of 1:2.5. Figure 6B reports the expression of the pLR1 RNA, as copy number per cell (black bars; plotted on the left axis). It can be observed that the higher amount of pLR1 RNA was on days 1 and 3, and decreased progressively on days 7 and 10, but gently augmented at day 14 and finally dropped at day 28. On days 21 and 28, many cells in the culture could be dead or in a pre-apoptotic stage, but some of them must be still viable and maintain the ability to transcribe DNA into RNA. The amount of pLR1 RNA determined is adjusted by the number of cells (quantified by the amount of total RNA, considering the average of 20 pg per cell). White bars (plotted on right axis) represent the luminescence intensity of the samples from the pLR1-transfected cells, which show a similar behavior. In this graph, a peak in the luminescence can be observed at day 3 and this fell progressively on days 7 and 10, increased at day 14 and lastly the luminescence disappeared at day 28. These results indicate that the luminescence was dependent on virus replication.

### 3.7. Functional Effect of the Adenovirus Genome Expression after AuNPs-Mediated Transfection

**Figure 7:**
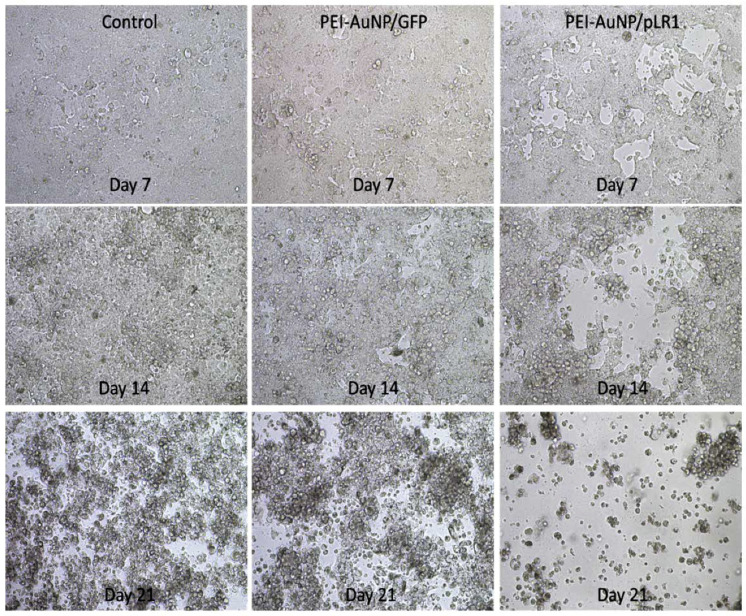
Oncolytic effect of PEI-AuNPs/pLR1. This figure shows the different microscope images at 10× of the SW480 cells taken at different days after transfections with PEI-AuNPs/GFP or PEI-AuNPs/pLR1 at 20 µg/mL of DNA. Control corresponds to non-transfected cells.

The generation of ICOVIR-15Luc was also evaluated by observing its cytopathic effect on cancer cells in culture. Figure 7 shows the microscopy images of SW480 cells taken 7, 14 and 21 days after transfection with PEI-AuNPs/pLR1 with a 1:2.5 weight ratio and a final concentration of 20 µg/mL. In the control images, we can observe some cell death on day 21 that could be due to maintaining the confluent cell culture for a long period of time. In cells transfected with tracer non-oncolytic PEI-AuNPs/p3C eGFP complexes, a slightly higher rate of cell death was observed probably due to the PEI cytotoxicity, since 72 h post-transfection, a viability loss of 30% compared with control cells was previously observed by the calcein esters test (data not shown). In contrast, in cells treated with PEI-AuNPs/pLR1, several empty areas were observed in the culture at day 7 and cells were completely lysed by day 21, probably due to the cytopathic effect of the oncolytic virus encoded by pLR1 plasmid.

## 4. Discussion

The main goal of this work was to achieve the synthesis and characterization of AuNPs that could carry large DNA plasmids, as those encoding oncolytic viruses, which is a very interesting tool in cancer therapy. For this purpose, AuNPs were functionalized in the presence of different concentrations of the cationic polymer PEI. PEI biodegradable polymer represents several advantages such as strong electrostatic interactions with DNA, oligonucleotide protection [47] and rapid DNA endosomal scape thanks to the “proton-sponge effect” [48]. PEI-AuNPs able to transport an oncolytic plasmid with the same size of adenovirus DNA were obtained. Theses nanoparticles bearing the DNA-coding oncolytic virus could transfect and destroy human tumor cells.

Firstly, we developed PEI-AuNPs with different Au core diameters (1.5–2.1 nm) and different total diameters (2.1–86.6 nm, hydrodynamic diameter). As expected, the nanoparticle size decreased as the concentration of PEI was increased due to different factors such as the presence of more PEI ligands or the interactions between the DNA and PEI chains. The nanoparticles synthesized with an initial PEI concentration of 8 mg/mL were selected for the transfection assays because, although they were slightly more cytotoxic, they had a smaller size with a higher DNA carrying capacity.

To find the suitable PEI-AuNPs/DNA ratio, DNA was added to PEI-AuNPs at different ratios. The size of PEI-AuNPs/DNA increased as the DNA amount increased. The PEI-AuNPs/DNA weight ratio of 1:2.5 was selected for further transfections. This PEI-AuNPs/DNA ratio could theoretically transport more DNA than the other ratios tested with a size of around 70 nm. This size is similar to that of the adenoviruses (70–90 nm) [49]. We observed that these PEI-AuNPs protected DNA from nuclease degradation and mediated as the effective transfection of p3C eGFP as PEI/DNA polyplexes. The cytotoxicity of transfection using PEI-AuNPs/DNA at 24, 48 and 72 h was very similar to that observed when using PEI/DNA polyplexes, and this suggests that toxicity is due to the amount of PEI employed [50,51] and not to the use of gold nanoparticles as vectors.

Once the transfection efficacy of the PEI-AuNPs was evaluated, we used them to transfect SW480 human colorectal cancer cells with pLR1 plasmid, which encodes the oncolytic adenovirus ICOVIR-15Luc. We cultured the transfected cells during 28 days and we observed the progressive oncolytic effect. A lysis effect was firstly appreciated at day 7 and all cells were lysed by day 21. We quantified the oncolytic plasmid RNA presence within the cultured cells and this decreased along the 28 days of sampling time with an increase peak at day 14. We also observed a little peak in luminescence on the same day, suggesting that the virus has been replicated at this time because the luminescence gene is regulated by the major late promoter of the virus. The levels of luminescence achieved when employing the pLR1 gene were low. This could be due to the mild activity of this promoter, lower than the CMV promoter present in pVK503TL plasmid. This fact and the high rate of cell lysis observed evidenced that the transfection of oncolytic plasmid in SW480 human cells was successful and the virus replicated in these cells. Cell lysis can be partially caused by cell culture and by PEI toxicity, but it is more evident in cells treated with the oncolytic adenovirus genome. Therefore, virus production is supported by late gene expression (luciferase expression driven by the major late promoter) and progressive cytopathic effect. The difference between a capsid-mediated virus infection and our plasmid delivery method involves the early phase of the virus life cycle that is the transfer of vDNA to the nuclei and the initiation of virus DNA replication. Later phases are independent of the genome delivery method. This has been demonstrated in plasmid-based methods to generate adenoviruses [52].

We show that PEI-AuNPs can carry a large DNA amount and effectively transfect cells “in vitro”. Further, the use of PEI-AuNPs showed to be useful to transfect tumor cells with the oncolytic plasmid pLR1. This approach could have interest in cancer treatment since it allows reducing the administration of oncolytic viruses, which is greatly limited by their immunogenicity and the pre-existing humoral immunity present in the general population [30,31,32]. Additional studies are needed to show the efficacy of these PEI-AuNPs/pLR1 “in vivo”.

## Figures and Tables

**Table 1:** Summary characterization of the gold-polyethylenimine (Au-PEI) nanoparticles (NPs) synthesized.

Au-NPs	Initial Concentration PEI (mg/mL)	Core Diameter TEM (nm)	Hydrodynamic Diameter Zetasizer (nm)	Zeta Potential (mV)	Molecules PEI/Np
**NP1**	1	2.1	86.6	39	2.1
**NP2**	2	1.8	6.5	35	3.8
**NP3**	4	1.6	5.5	25	6.4
**NP4**	8	1.5	2.1	34	9.4

**Table 2:** Summary characterization of NP4/DNA complexes with different amounts of DNA.

Au-NPs	PEI-AuNP/DNA Ratio (µg/µg)	Core Diameter TEM (nm)	Hydrodynamic Diameter Zetasizer (nm)	Zeta Potential (mV)
**NP4**	1:0	1.5	2.1	34
	1:0.01	2.1	5.66	4.33
	1:0.07	2.1	6.97	11.67
	1:0.3	2.1	37.21	14.67
	1:0.6	2.1	49.06	25
	1:1.25	2.1	38.18	18.67
	1:2.5	2.1	66.78	27
